# Transcutaneous electrical diaphragmatic stimulation in mechanically ventilated patients: a randomised study

**DOI:** 10.1186/s13054-023-04597-1

**Published:** 2023-08-30

**Authors:** Clément Medrinal, Margaux Machefert, Bouchra Lamia, Tristan Bonnevie, Francis-Edouard Gravier, Roger Hilfiker, Guillaume Prieur, Yann Combret

**Affiliations:** 1 Université Paris-Saclay, UVSQ, Erphan, 78000 Versailles, France; 2 Intensive Care Unit Department, Le Havre Hospital, Avenue Pierre Mendes France, 76290 Montivilliers, France; 3 Physiotherapy Department, Le Havre Hospital, Avenue Pierre Mendes France, 76290 Montivilliers, France; 4 Normandie Univ, UNIROUEN, EA3830-GRHV, 76 000 Rouen, France; 5 Institute for Research and Innovation in Biomedicine (IRIB), 76 000 Rouen, France; 6 Pulmonology Department, Le Havre Hospital, Avenue Pierre Mendes France, 76290 Montivilliers, France; 7 Pulmonology, Respiratory Department, Rouen University Hospital, Rouen, France; 8 Adir Association, 76230 Bois Guillaume, France; 9 Research and Independent Studies in Private Physiotherapy (RISE), 3902 Brig, Switzerland

**Keywords:** Diaphragm dysfunction, Electrical stimulation, Intensive care unit, Mechanical ventilation

## Abstract

**Background:**

Few specific methods are available to reduce the risk of diaphragmatic dysfunction for patients under mechanical ventilation. The number of studies involving transcutaneous electrical stimulation of the diaphragm (TEDS) is increasing but none report results for diaphragmatic measurements, and they lack power. We hypothesised that the use of TEDS would decrease diaphragmatic dysfunction and improve respiratory muscle strength in patients in ICU.

**Methods:**

We conducted a controlled trial to assess the impact of daily active electrical stimulation versus sham stimulation on the prevention of diaphragm dysfunction during the weaning process from mechanical ventilation. The evaluation was based on ultrasound measurements of diaphragm thickening fraction during spontaneous breathing trials. We also measured maximal inspiratory muscle pressure (MIP), peak cough flow (PEF) and extubation failure.

**Results:**

Sixty-six patients were included and randomised using a 1:1 ratio. The mean number of days of mechanical ventilation was 10 ± 6.8. Diaphragm thickening fraction was > 30% at the SBT for 67% of participants in the TEDS group and 54% of the Sham group (OR1.55, 95% CI 0.47–5.1; *p* = 0.47). MIP and PEF were similar in the TEDS and Sham groups (respectively 35.5 ± 11.9 vs 29.7 ± 11.7 cmH_2_0; *p* = 0.469 and 83.2 ± 39.5 vs. 75.3 ± 34.08 L/min; *p* = 0.83). Rate of extubation failure was not different between groups.

**Conclusion:**

TEDS did not prevent diaphragm dysfunction or improve inspiratory muscle strength in mechanically ventilated patients.

*Trial registration*: Prospectively registered on the 20th November 2019 on ClinicalTrials.gov Identifier NCT04171024.

**Supplementary Information:**

The online version contains supplementary material available at 10.1186/s13054-023-04597-1.

## Background

Inspiratory muscle weakness is caused by the suppression of respiratory muscle activity by sedative agents and mechanical ventilation, as well as other inflammatory mechanisms [1–3]. Diaphragmatic dysfunction is now widely described in patients under mechanical ventilation in intensive care units (ICU). In a recent meta-analysis, we found that the prevalence of diaphragmatic dysfunction at the time of the spontaneous breathing trial ranged from 20% to over 60% and was strongly associated with an increased risk of extubation failure and death [4]. Rehabilitation is commonly performed in the ICU, but few specific methods are available to reduce the risk of diaphragmatic dysfunction. Inspiratory muscle training is one of the most studied methods and seems to increase the maximal inspiratory pressure (MIP) [5]. However, the heterogeneity of the protocols, the equipment used and the selection of the participants reduce the certainty of the clinical benefits. Moreover, by its mechanism of action, this form of rehabilitation requires people to be able to breath spontaneously and to cooperate [6].

Neuromuscular electrical stimulation can be used to maintain muscle thickness and strength and has the advantage of not requiring cooperation since it produces involuntary muscle contractions. Data are mostly available for the lower limb muscles, particularly the quadriceps [7–9]. However, the number of studies involving transcutaneous electrical stimulation of the diaphragm (TEDS) is increasing [10–18]. TEDS provides non-invasive stimulation through surface electrodes placed bilaterally on the thorax over the diaphragm apposition zone. Studies in people on mechanical ventilation report good feasibility and safety of this method [10, 13, 15–18]. For patients on mechanical ventilation, TEDS is reported to increase the number of type II fibres [19], increase MIP and maximal expiratory pressure (MEP) and decrease the rate of ventilatory weaning failure [15, 16, 18]. However, none of these studies specifically measured the diaphragm function, and the samples were small with no blinding of the main outcome. We hypothesised that the use of TEDS would decrease diaphragmatic dysfunction and improve respiratory muscle strength in people in ICU. The primary aim of this study was to assess the effectiveness of TEDS in reducing the number of people with diaphragmatic weakness as assessed by diaphragm thickening at the time of weaning from mechanical ventilation.

## Method

### Design and recruitment

We conducted a single centre, double-blind randomised controlled trial with intention to treat analysis in our 18 bed ICU at Le Havre Hospital in France. We recruited consecutive individuals with a diagnosis that did not involve covid-19, who were admitted to our ICU between December 2019 and July 2022. The inclusion criteria were aged > 18 years, ventilated for at least 24 h with an expected stay of more than 72 h in the unit. The patients were required to be fully independent prior to admission to the ICU. The exclusion criteria were having been hospitalised for > 72 h before ICU admission, having a pacemaker or an implantable defibrillator, a cutaneous lesion that could interfere with probes, neurological pathology with disabling muscle weakness, chronic loss of autonomy (defined by a Katz score below 6/6), BMI > 35 kg/m^2^, severe COPD (FEV1 < 30%) and a decision to withhold life-sustaining treatment.

Ethical approval was granted by the French Comité de Protection des Personnes Ile de France X (38-19). All participants or their relatives provided written informed consent for participation. This study was prospectively registered (NCT04171024) and is reported according to the CONSORT guidelines.

Randomisation was performed by a computer-generated random number sequence (http://www.edgarweb.org.uk/) with concealed allocation. Participants were allocated to either receive usual care and sham electrical stimulation (Sham Group) or usual care and a transcutaneous electrical diaphragm stimulation (TEDS Group).

### Procedure

The medical, paramedical and physiotherapist teams involved in the care and decision to wean the patient from mechanical ventilation were not aware of the participant’s group allocation. Each morning, after the daily assessment for contraindications to transcutaneous electrical stimulation and diaphragm thickness measurement, blind investigators (CM and YC) positioned the electrodes according to the ultrasound location (see Additional file 1.). Then, the physiotherapists (MM and GP) applied Sham or TEDS stimulation. Each morning, two pairs of electrodes (5 × 5 cm) were applied to each hemithorax; one pair above and one pair below the sides of the xiphoid process between the 8th and 10th anterior intercostal spaces. The second pair was applied over the medio-axillary line of the thorax between the 8th and 10th intercostal spaces [10, 15].

The stimulation applied to the TEDS group was a bidirectional current with a frequency of 50 Hz and an impulse width of 300 ms. The intensity was set to produce a palpable contraction of the muscles under the probes [17]. Each cycle was programmed to produce 6 s of stimulation and 10 s of rest. Cycles were not completely synchronised with inspiration and could be applied with both controlled mode ventilation and pressure support mode. The treatment was performed daily, 5 times per week. The electrical stimulation session lasted 20 min. The stimulation was not applied when the participant was under curare, in a prone position, hemodynamically unstable despite catecholamines, or agitated (Ramsay sedation score 1/6).

The sham stimulation was a bidirectional current with a frequency of 2 Hz and a pulse duration of 300 microseconds; this did not produce muscle contractions but created some interference with the monitoring signals to ensure blinding of the nursing staff. The sham session lasted 20 min. TEDS or SHAM stimulation was applied until the first extubation attempt.

The care and weaning protocols were standard practice on the ward and were identical between participants. The physicians who decided on patient care and ventilatory weaning were not aware of the randomisation or the daily diaphragmatic ultrasound measurements.

### Measures

#### Primary aim

Every morning, between 10 and 11 a.m., we used the Philips CX 50 ultrasound machine with a linear probe (5–12 MHz) to measure diaphragm thickness. The probe was placed perpendicular to the skin in the zone of apposition between the mid-axillary or antero-axillary line, in the 8th to 11th intercostal spaces. We measured diaphragm thickness perpendicular to the direction of the fibres between the pleural and peritoneal membranes, but not including the membranes. As previously described [20, 21], diaphragm thickening fraction (DTF) (end-inspiratory thickness−end-expiratory thickness)/end-expiratory thickness × 100% was measured by one of two blind assessors (CM and YC), after the decision to extubate that was taken at the start of the spontaneous breathing trial (SBT) (Pressure support mode with Inspiratory pressure at 10 cmH20 and Positive Expiratory pressure at 0 cmH20).

If the SBT failed, and the decision to extubate was delayed, the value used for the analysis was that measured on the day of the extubation. Based on the results of our meta-analysis on the predictive values of the DTF on weaning failure, we defined diaphragmatic dysfunction as a DTF < 30% and considered a DTF of 20% to indicate severe dysfunction [4].

#### Secondary aims

##### Respiratory muscle evaluation

The blind assessors (CM and YC) monitored daily changes in end-tidal thickness of the right hemidiaphragm from the day of inclusion until extubation using a high frequency (13 MHz) linear transducer over the apposition zone to monitor diaphragm thickness. Maximum inspiratory pressure (MIP) and cough peak expiratory flow (PEF) were also measured at the beginning of the SBT. The MIP was measured using an electronic manometer with a micro-RPM® unidirectional valve. Participants were informed that MIP would be evaluated at the residual volume and were instructed accordingly. The participant was disconnected from the ventilator for a minimum of 20 s [22]. PEF was recorded by the flow curve of the ventilator. The participant was asked to inspire maximally before coughing. Three measurements of MIP and PEF were carried out and the best were used in the analysis.

##### Characteristics and clinical outcomes

At admission, we collected demographic data, comorbidities, primary cause of admission and severity of illness by Simplified Acute Physiology Score (SAPS 2) and Sequential Organ Failure Assessment (SOFA). Each day we collected ventilator setting, administration of neuromuscular blockers or sedative drugs and Ramsay sedation score. From the day of intubation until the first extubation or death, we counted events such as SBT failure (defined as the clinical impossibility to extubate the patient and re-establish initial ventilatory parameters), extubation and extubation failure (defined as re-intubation within 48 h or death), causes of extubation failure, tracheostomy and discharge from the ICU. Ventilator-free days were computed to 28 days. (Participants who required more than 28 days of ventilatory support or who died before 28 days were counted as 0.)

### Statistical analysis

Based on the results of the studies with the most robust methodology [23–25], we estimated a rate of diaphragm dysfunction at weaning at 60% in the control group. Considering the response rate to diaphragm stimulation according to previous studies, we estimated a 60% response rate in the TEDS group. Therefore, a sample size of 56 participants was required for a statistical power of 80% and an alpha level at 0.05. Anticipating a dropout rate of 20% we planned to include 66 individuals.

Baseline data for both groups were summarised using means and standard deviations for continuous variables and absolute and relative frequencies for categorical data. We performed a chained equation multiple imputation with 20 imputed datasets for missing data.

The between group differences were modelled using linear regression for quantitative variables and expressed as mean differences and 95% confidence intervals. For these between group differences, a Cohen’s *d* effect size was calculated. The binary response variables were modelled with logistic regression and expressed as odds ratios and 95% confidence intervals. We ran diagnostic tests for the regression analyses, and we decided to log transform the diaphragm thickening fraction data. The number of spontaneous breathing trial (SBT) failures, the time to extubation and the number of days without mechanical ventilation at 28 days were analysed using a cox-regression and presented with hazard ratios and 95% confidence intervals. Results were adjusted by a predefined variable: The diaphragm thickness at the start of the study [26]. The Pearson r coefficient was used to calculate the correlation between diaphragm thickening fraction and maximal inspiratory pressure. Two-tailed p-values were calculated for all results and considered significant if < 0.05.

All statistical analyses were performed with Stata version 17.1 (StataCorp LLC, College Station, Texas, USA).

## Results

**Fig. 1:**
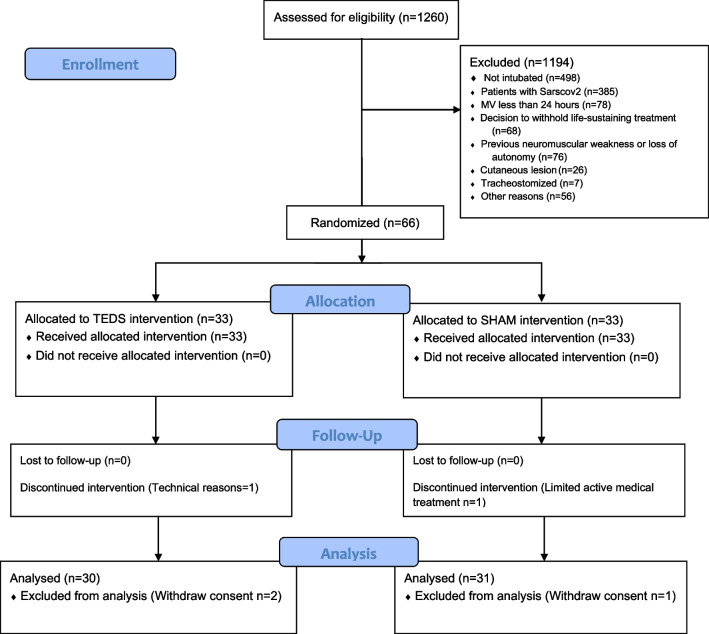
Flowchart of the study

In total, 1260 individuals were admitted to our ICU between December 2019 and July 2022. Of these, 498 did not receive invasive mechanical ventilation and 385 had a diagnosis of COVID-19. The remaining 384 were screened for eligibility and 66 were included and randomised (Fig. 1). During the study, 1 person was excluded from the analysis due to technical issues, 1 was on limited active medical treatment and 3 withdrew their consent. Table 1 presents participant characteristics. Briefly, participants were mostly male (77%), mean (SD) age of 62 (13) years, mean BMI 27.5 (5.5) and mean SAPS 2 at inclusion 29 (12), with no between group differences except for a higher rate of previous neurological events in the TEDS group but with no functional sequalae.

**Table 1:** Cohort characteristics

Variables	Total	TEDS group	Sham group	Mean or risk difference with 95% CI
	(*N* = 61)	(*N* = 30)	(*N* = 31)	
*At admission*				
Age, years	62 (13)	62 (10)	61 (15)	1.5 (− 5.0 to 8.1)
Gender (female)	15 (23%)	11 (33%)	4 (12%)	0.02 (− 0.05 to 0.49)
Height, cm	169 (9.1)	168 (10)	171 (7.9)	− 2.6 (− 7.3 to 2.1)
Weight, kg	79 (17)	78 (15)	80 (19)	− 2.3 (− 11 to 6.5)
Body mass index, kg/m^2^	27 (5.5)	27 (5.1)	27 (5.9)	0.06 (− 2.8 to 2.9)
SAPS2 at ICU admission, ua	29 (12)	28 (13)	30 (12)	− 1.5 (− 8.0 to 5.0)
SOFA score at ICU admission, ua	9.3 (3.4)	9.1 (3.7)	10 (3.2)	− 0.46 (− 2.3 to 1.4)
Diaphragm thickness, cm	0.19 (0.04)	0.194 (0.04)	0.186 (0.04)	0 (− 0.01 to 0.01)
*Main cause of admission*				
Pneumonia	13 (22%)	4 (14%)	9 (29%)	− 0.15 (− 0.36 to 0.05)
COPD/asthma exacerbation	8 (13%)	4 (14%)	4 (13%)	0.01 (− 0.16 to 0.18)
Cardiac failure	5 (8.3%)	4 (14%)	1 (3.2%)	0.11 (− 0.03 to 0.25)
Cardiac arrest	4 (6.7%)	1 (3.4%)	3 (10%)	− 0.06 (− 0.19 to 0.06)
Drug overdose	1 (1.7%)	1 (3.4%)	0 (0.00%)	0.03 (− 0.03 to 0.10)
Acute mental status change	6 (10%)	4 (14%)	2 (6.5%)	0.07 (− 0.08 to 0.23)
Shock	9 (15%)	3 (10%)	6 (19%)	− 0.09 (− 0.27 to 0.09)
Intra-abdominal sepsis with surgery	9 (15%)	6 (21%)	3 (10%)	0.11 (− 0.07 to 0.29)
*Co-morbidity*				
Chronic respiratory diseases	18 (30%)	7 (24%)	11 (35%)	− 0.11 (− 0.34 to 0.12)
Chronic cardiac insufficiency	17 (28%)	9 (31%)	8 (26%)	0.05 (− 0.18 to 0.28)
Hypertension	27 (45%)	16 (55%)	11 (35%)	0.20 (− 0.05 to 0.44)
Chronic kidney insufficiency	3 (5.0%)	1 (3.4%)	2 (6.5%)	− 0.03 (− 0.14 to 0.08)
Diabetes	18 (30%)	10 (34%)	8 (26%)	0.09 (− 0.14 to 0.32)
Neurological previous story	7 (12%)	6 (21%)	1 (3.2%)	0.17 (0.01 to 0.33)
Dyslipidaemia	9 (15%)	6 (21%)	3 (10%)	0.11 (− 0.07 to 0.29)
Obesity	14 (23%)	8 (28%)	6 (19%)	0.08 (− 0.13 to 0.30)
*Between admission and awakening*				
Ventilator acquired Pneumonia	11 (18%)	4 (14%)	7 (23%)	− 0.09 (− 0.28 to 0.11)
Sepsis	26 (43%)	12 (41%)	14 (45%)	− 0.04 (− 0.29 to 0.21)
Multi organ failure	12 (20%)	7 (24%)	5 (16%)	0.08 (− 0.12 to 0.28)
Prone positioning	6 (10%)	3 (10%)	3 (10%)	0.01 (− 0.15 to 0.16)
Dialysis	8 (13%)	3 (10%)	5 (16%)	− 0.06 (− 0.23 to 0.11)
Use of neuromuscular blockers	14 (23%)	8 (28%)	6 (19%)	0.08 (− 0.13 to 0.30)
N°days of neuromuscular blockers	1.8 (4.0)	1.6 (2.7)	1.9 (5.0)	− 0.36 (− 2.5 to 1.8)
N°days under sedative agent	6.5 (5.8)	6.2 (4.5)	6.8 (6.9)	− 0.59 (− 3.7 to 2.5)
N°days under mechanical ventilation	10 (6.8)	8.6 (4.7)	11 (8.3)	− 2.1 (− 5.7 to 1.5)
N°days of assist controlled MV	5.5 (5.6)	5.1 (3.8)	5.8 (6.9)	− 0.69 (− 3.7 to 2.3)
N°days of pressure support MV	4.2 (5.0)	3.4 (4.0)	4.9 (5.7)	− 1.4 (− 4.0 to 1.2)
Use of corticosteroids	11 (18%)	6 (21%)	5 (16%)	0.05 (− 0.15 to 0.24)

Data are expressed at mean (SD) and at *n* (%)

CI, confidence interval; SD, standard deviation; SAPS, simplified acute physiology score; SOFA, sequential organ failure assessment; ICU, intensive care unit; ua, unity arbitrary; COPD, chronic pulmonary disease; MV, mechanical ventilation

**Fig. 2:**
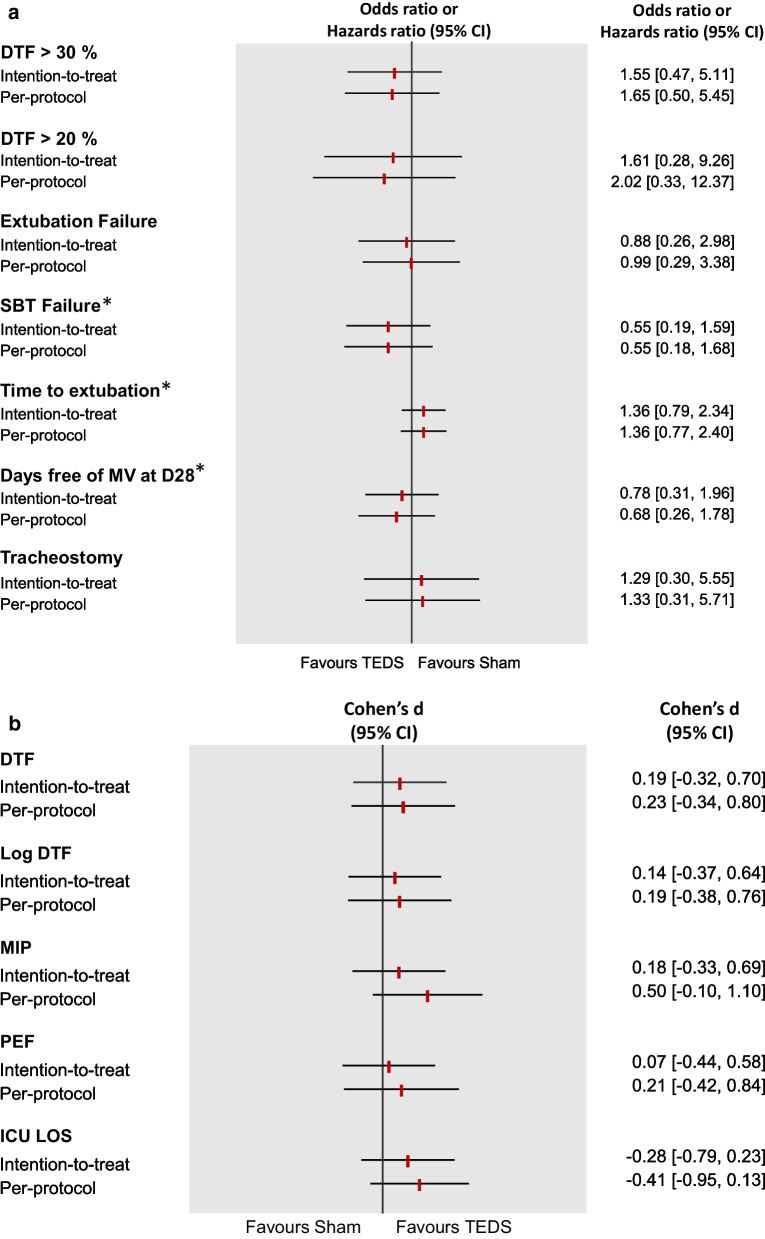
**a** Comparison between intention to treat and per protocol analysis for dichotomic outcomes. Data are as odds ratios and 95% confidence intervals. *Data are hazard ratios and 95% confidence intervals. CI, confidence interval; DTF, diaphragm thickening fraction; SBT, spontaneous breathing trial; MV, mechanical ventilation; D28, 28 days. **b** Comparison between intention to treat and per protocol analysis for continuous outcomes. MIP, maximal inspiratory pressure; PEF, peak cough flow; ICU LOS, intensive care unit length of stay

No adverse event was reported during the sessions. The TEDS and sham procedure started, respectively, after 40 (36) versus 38 (57) hours; *p* = 0.29. Participants in the TEDS group received diaphragmatic electrical stimulation for a mean 3.7 (2.2) days against 4.7 (4) for the SHAM group and the mean intensity was 48 (9.5) mA. On the day of extubation, diaphragm thickening fraction was > 30% for 67% of participants in the TEDS group and 54% of the Sham group (respectively OR 1.65, 95% CI 0.5–5.39; *p* = 0.41 and OR 1.55, 95% CI 0.47–5.1; *p* = 0.47 on the ITT). ITT and per protocol analyses yielded similar results (Fig. 2). The comparison between the adjusted and non-adjusted analysis is shown in Additional files 2 and 3.

On the day of extubation, diaphragm thickening fraction was > 20% for 92% of the TEDS group and 83% of the sham group (OR 1.61, 95% CI 0.28–9.4; *p* = 0.59).

**Fig. 3:**
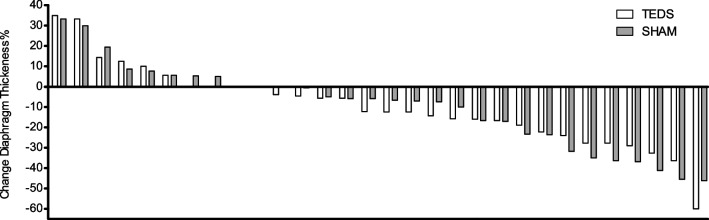
Individual percentage of change in diaphragm thickness during the study between TEDS and Sham Groups. The change was calculated as the difference between the first and last days

The mean loss of diaphragm thickness during mechanical ventilation did not differ between groups (respectively 9.6%, 95% CI 2.2–17 vs. 9.6%, 95% CI 1.8–17.4; *p* = 0.99) (Fig. 3). The time course of diaphragm atrophy according to the mechanical ventilation mode (active cycling or pressure support) is presented in Additional file 4. Patients whose thickness decreased by at least at 10% received more days of mechanical ventilation (11.3 ± 6.4 vs 7.8 ± 6.8 days; *p* = 0.002.)

DTF and MIP during the SBT were moderately correlated (*r* = 0.4, 95% CI 0.12–0.61; *p* < 0.01), however DTF and MIP were similar in the TEDS and SHAM group. We found no other between group differences in respiratory muscle variables or the secondary outcomes (Table 2).

**Table 2:** Comparison between TEDS and Sham group according to per protocol or intention to treat analysis

	TEDS group	Sham group	Per protocol analysis		Intent to treat analysis		Cohen’ *d*
			Adjusted analysis		Adjusted analysis		
			Estimate (95% CI)	*p* value	Estimate (95% CI)	*p* value	
Primary outcome							
DTF > 30%, *n* (%)	16.0 (66.7%)	13.0 (54.2%)	1.65 (0.50 to 5.39)	0.411	1.55 (0.47 to 5.10)	0.472	–
DTF > 20%, *n* (%)	22.0 (91.7%)	20.0 (83.3%)	2.02 (0.33 to 12.40)	0.449	1.61 (0.28 to 9.25)	0.591	–
Secondary outcomes							
DTF (%), mean (SD)	47.46 (34.19)	38.67 (23.90)	6.46 (− 10.19 to 23.11)	0.438	6.86 (− 11.03 to 24.75)	0.442	0.19 (− 0.32 to 0.70)
Log DTF (%), mean (SD)	3.65 (0.64)	3.50 (0.58)	0.11 (− 0.24 to 0.47)	0.531	0.12 (− 0.32 to 0.56)	0.585	0.14 (− 0.37 to 0.64)
MIP cmH20, mean (SD)	35.57 (11.90)	29.71 (11.15)	5.83 (− 1.53 to 13.19)	0.117	2.77 (− 4.87 to 10.41)	0.469	0.18 (− 0.33 to 0.69)
PEF (L/min), mean (SD)	83.20 (39.57)	75.37 (34.08)	7.80 (− 16.59 to 32.19)	0.521	3.58 (− 21.78 to 28.94)	0.776	0.07 (− 0.44 to 0.82)
Extubation failure, *n* (%)	7.0 (29.2%)	8.0 (32.0%)	0.99 (0.29 to 3.40)	0.981	0.88 (0.26 to 3.00)	0.836	–
SBT failure, median (IQR)	0.0 (0.0 to 1.0)	1.0 (0.0 to 1.0)	0.55 (0.18 to 1.64)	0.283	0.55 (0.19 to 1.56)	0.259	–
Time to extubation, median (IQR)	8.0 (5.0 to 10.5)	8.5 (5.0 to 13.5)	1.36 (0.77 to 2.38)	0.288	1.36 (0.79 to 2.34)	0.262	–
Days free of MV at d 28, median (IQR)	19.5 (4.0 to 22.5)	21.0 (13.0 to 23.0)	0.68 (0.26 to 1.76)	0.430	0.78 (0.31 to 1.92)	0.583	–
Tracheostomy, *n* (%)	5.0 (18.5%)	4.0 (15.4%)	1.33 (0.31 to 5.72)	0.702	1.29 (0.30 to 5.48)	0.729	–
ICU lOS, mean (SD)	14.89 (9.52)	18.21 (12.83)	− 4.60 (− 10.93 to 1.73)	0.151	− 3.40 (− 9.43 to 2.63)	0.264	− 0.28 (− 0.79 to 0.23)

CI, confidence interval; DTF, diaphragm thickening fraction; SD, standard deviation; MIP, maximal inspiratory pressure; PEF, peak expiratory flow; SBT, spontaneous breathing trial; MV, mechanical ventilation; ICU, intensive care unit; LOS, length of stay

## Discussion

This study is the first to compare the use of transcutaneous electrical stimulation of the diaphragm with a sham intervention on diaphragm function in intubated and ventilated patients in the ICU. We found that: (1) TEDS did not significantly decrease the risk of diaphragm dysfunction; (2) both groups had similar decreases in diaphragmatic thickness during ventilation; (3) TEDS would not result in a statistically significant improvement in inspiratory muscle strength or peak expiratory flow during cough and (4) TEDS would not optimise weaning from mechanical ventilation and extubation success rate.

Limiting the adverse effects of mechanical ventilation on the diaphragm is challenging but essential because these effects lead to poor outcomes. ICU teams are seeking new methods to maintain a sufficient level of diaphragm activity while also maintaining lung-protective ventilation and avoiding excessive respiratory effort [27, 28]. In this context, we hypothesised that TEDS could maintain sufficient muscle thickness and prevent the development of ventilation-induced diaphragm dysfunction. Several teams have studied the effects of transvenous phrenic nerve stimulation showing promising results on inspiratory muscle strength [29, 30]. Nerve stimulation allows the recruitment of the entire diaphragm, which was not the case in our study as no ventilatory cycle was generated by the period of electrical stimulation. This stimulation did not reduce muscle atrophy, as evidenced by the fact that both groups experienced a decrease in diaphragmatic thickness of approximately 10%. Additionally, 40% of the total sample exhibited a decrease of 10% or more. We suggest three explanations for these results. First, transcutaneous electrical stimulation only allows contraction of the muscle fibres located under the electrode; second, the contraction of the muscle fibres did not generate a movement of the diaphragm muscle; and third, the duration was only twenty minutes. This situation is superimposed on the stimulation of the limb muscles [31, 32]. Thus, this stimulation can induce a superficial muscle contraction, but the intensity applied does generate movement, reflecting less effectiveness than a voluntary movement produced against resistance. The number of publications relating to stimulation is currently increasing. This technique has been reported to induce changes in diaphragmatic muscle fibre type in rats [19], be safe for use in patients on mechanical ventilation and to improve ventilatory and muscle function [10, 18]. However, none report results on diaphragmatic measurements, and they lacked power. Our daily diaphragm measurements, as well as the measurement of the diaphragm thickening fraction invalidates previous hypotheses for strong benefits. However, TEDS may improve intercostal muscle function [33]. Indeed, the visible intercostal muscle contractions during stimulation and the tendency towards an improvement in MIP may indicate an increase in the strength of the accessory inspiratory muscles. If this is the case, TEDS could allow early intervention with no requirement of patient cooperation. Our evaluation focused on the respiratory muscles. As our intervention specifically targeted these muscles, we did not report data on acquired limb muscle weakness during intensive care, which could have influenced the results. However, all intubated patients in our department receive daily physiotherapy with experienced therapists who adjust the level of exercise according to the patient’s level of consciousness and muscle strength.

Our study has several limitations. First, the attrition rate was greater than we had estimated. Although we included 20% more participants than required according to the sample size calculation, we had 27% missing data. However, the intention-to-treat and per-protocol analysis results did not differ and reached the same conclusions. Also, there was no difference between participants who dropped out and those who were analysed. Second, although the diaphragm thickening fraction is accepted as an indicator of diaphragm function and a predictor for extubation failure, some recent data suggest that the correlation between ultrasonography and diaphragm pressure output is unsatisfactory, is highly variable and depends on body position [34]. Furthermore, there is no consensus on the choice of cutoff value [20]. Although we chose two values (< 20% and < 30%) for our analyses, and we standardised the time of the DTF measurement and the ventilatory and neurological conditions of the participants, we did not control for other factors that could influence respiratory drive at the time of the SBT, such as the measurement of perceived dyspnoea [35, 36]. It would be better to measure diaphragm dysfunction by phrenic magnetic nerve stimulation but when we designed our study we standardised our diaphragm measurement and one paper demonstrated a strong correlation between magnetic stimulation and diaphragm thickening fraction during spontaneous breathing with pressure support [37]. TEDS was not completely synchronised with inspiration because (1) the inspiratory time is short thereby needing a steep increase in the current intensity, and (2) there is currently no device that allows this synchronisation. It is important to exercise caution when using this type of stimulation because unsynchronised stimulation can lead to eccentric contractions of the diaphragm during the expiratory phase, and the potential risks associated with it are not yet well determined [38, 39]. We recognise that the external validity of our results may be limited. The study was single centre with a small sample size, and our department protocols may differ from those in other centres. TEDS was applied partially to spontaneously breathing patients, which indeed makes it more challenging to interpret its potential in the setting of diaphragm inactivity. Additionally, the study sample was highly selected, and the results may have differed in different conditions or with the addition of other strengthening methods, such as inspiratory muscle training.

Finally, the stimulation induced noise in the cardiac monitoring data, which may disturb monitoring processes by other clinicians (see Additional file 5.).

## Conclusion

This study is the first to investigate the effect of TEDS on diaphragm function in ventilated critically ill patients and to compare it with a sham intervention. TEDS did not prevent diaphragm dysfunction or muscle atrophy. Clinically important outcomes were not better with TEDS. Further studies are warranted to address these previous limitations and develop more robust and efficient stimulation approaches.

### Supplementary Information

**Additional file 1.** Ultrasound localisation and electrode position. Philips CX 50 ultrasound machine with a linear probe (5–12 MHz) to measure diaphragm thickness. The probe was placed perpendicular to the skin in the zone of apposition between the mid-axillary or antero-axillary line, in the 8th to 11th intercostal spaces. The red cross indicates the diaphragm muscle fibres located between the upper and lower membranes.

**Additional file 2.** Per protocol analysis with and without adjustment

**Additional file 3.** Intention to treat analysis with and without adjustment

**Additional file 4.** The time course of diaphragm atrophy according to the mechanical ventilation mode during the first 21 days. CMV: Controlled Mechanical Ventilation mode; PS: Pressure Support Ventilation mode.

**Additional file 5.** Possible noises observed with cardiac monitoring during the stimulation periods. "Active phase" corresponds to the periods of electrical stimulation.

## Data Availability

The datasets used and/or analysed during the current study are available from the corresponding author on reasonable request.
