# Supplementary figures and images for: Transcutaneous electrical diaphragmatic stimulation in mechanically ventilated patients: a randomised study
Source: Crit Care. 2023 Aug 30;27:338. doi: 10.1186/s13054-023-04597-1 (PMC10469422; doi:10.1186/s13054-023-04597-1)

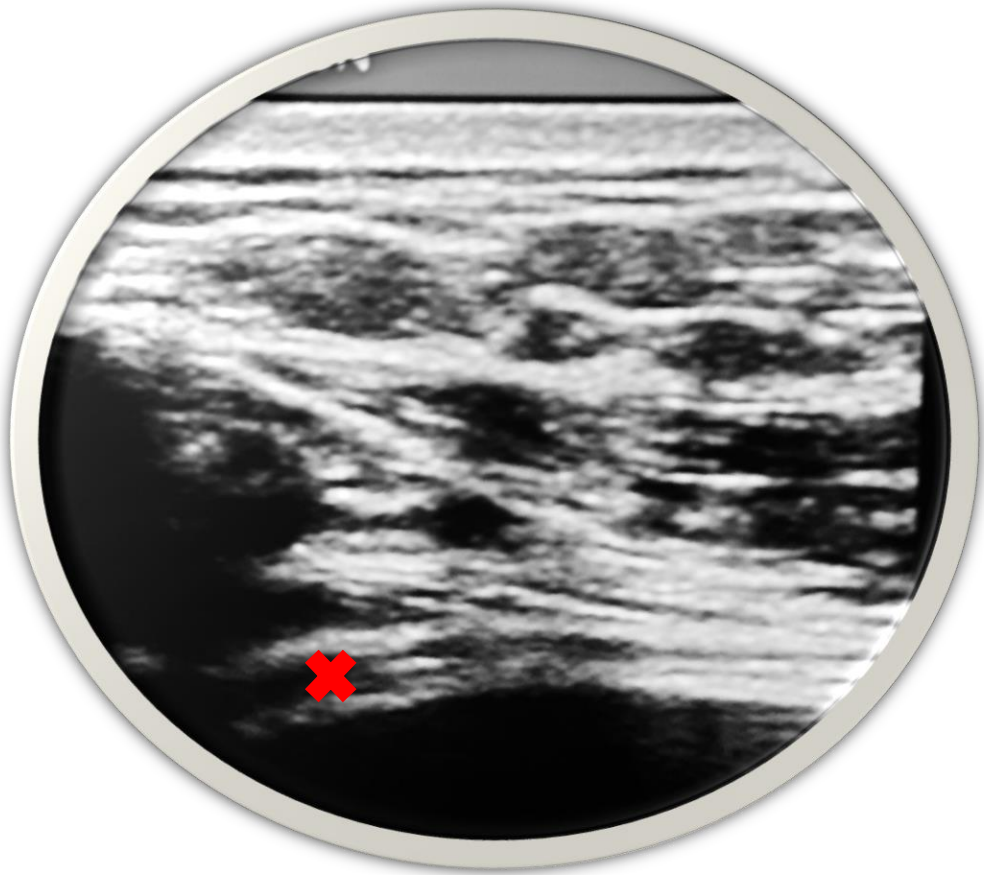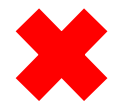

Diaphragm

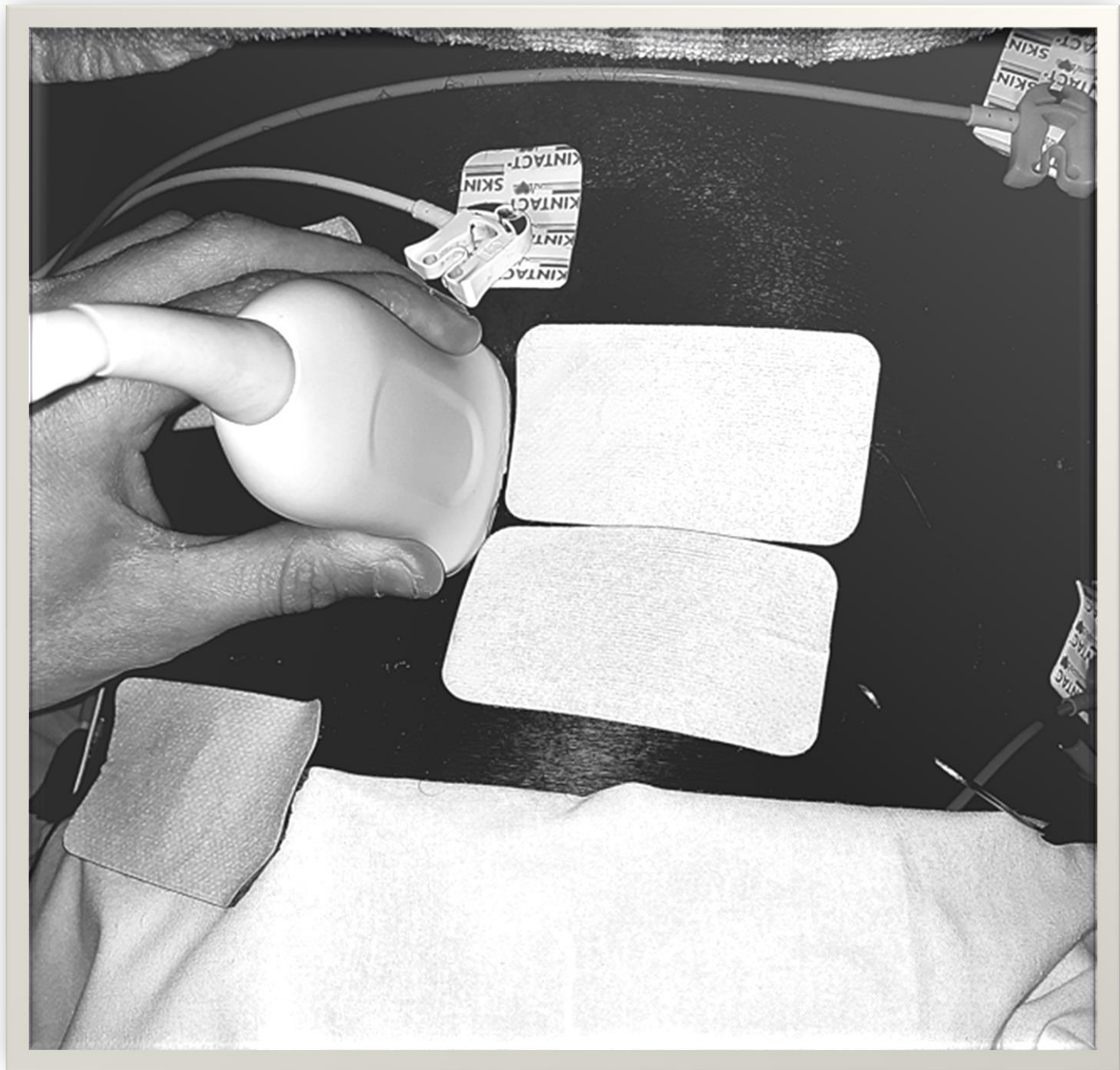

Supplement: Supplementary file 1 — Additional file 1. Ultrasound localisation and electrode position. Philips CX 50 ultrasound machine with a linear probe (5–12 MHz) to measure diaphragm thickness. The probe was placed perpendicular to the skin in the zone of apposition between the mid-axillary or antero-axillary line, in the 8th to 11th intercostal spaces. The red cross indicates the diaphragm muscle fibres located between the upper and lower membranes. [file 13054_2023_4597_MOESM1_ESM.pdf]

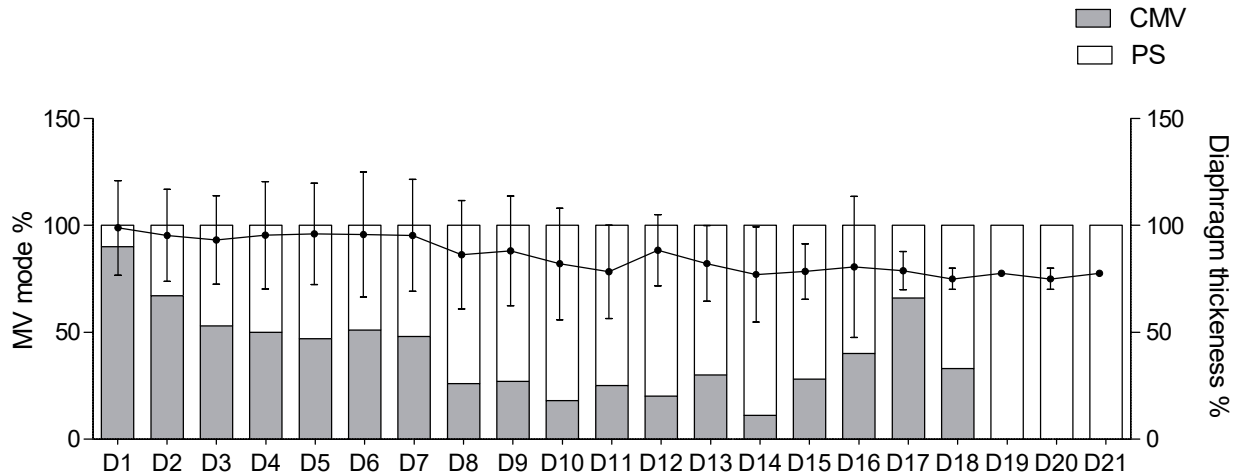

Supplement: Supplementary file 4 — Additional file 4. The time course of diaphragm atrophy according to the mechanical ventilation mode during the first 21 days. CMV: Controlled Mechanical Ventilation mode; PS: Pressure Support Ventilation mode. [file 13054_2023_4597_MOESM4_ESM.pdf]

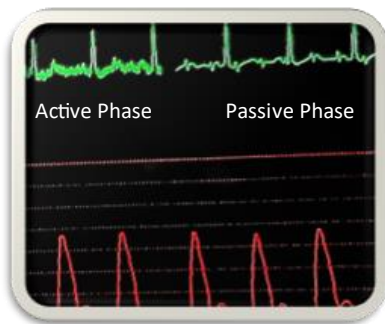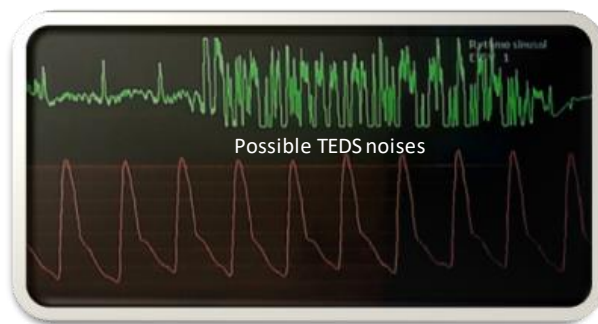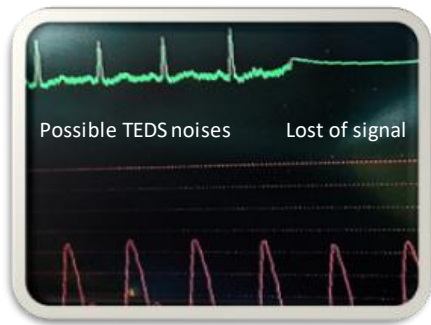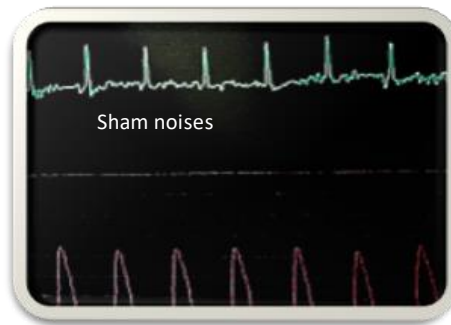

Supplement: Supplementary file 5 — Additional file 5. Possible noises observed with cardiac monitoring during the stimulation periods. "Active phase" corresponds to the periods of electrical stimulation. [file 13054_2023_4597_MOESM5_ESM.pdf]
