# Supplementary material for: Transcutaneous electrical diaphragmatic stimulation in mechanically ventilated patients: a randomised study
Source: Crit Care. 2023 Aug 30;27:338. doi: 10.1186/s13054-023-04597-1 (PMC10469422; doi:10.1186/s13054-023-04597-1)
Supplement: Supplementary file 2 — Additional file 2. Per protocol analysis with and without adjustment [file 13054_2023_4597_MOESM2_ESM.docx]

ESM 1 Per protocol analysis with and without adjustement

|  |  |  | Non adjusted Per Protocol Analysis | | Adjusted analysis Per Protocol | |
| --- | --- | --- | --- | --- | --- | --- |
|  | TEDS Group | Sham Group | Estimate (95%CI) | p-value | Estimate (95%CI) | p-value |
| Primary outcome |  |  |  |  |  |  |
| DTF>30%, n (%) | 16.0  (66.7%) | 13.0  (54.2%) | 1.69  (0.53 to 5.44) | 0.377 | 1.65  (0.50 to 5.39) | 0.411 |
| DTF>20%, n (%) | 22.0  (91.7%) | 20.0  (83.3%) | 2.20  (0.36 to 13.34) | 0.391 | 2.02  (0.33 to 12.40) | 0.449 |
| Secondary outcomes |  |  |  |  |  |  |
| DTF (%), mean (SD) | 47.46  (34.19) | 38.67  (23.90) | 8.79  (-8.35 to 25.93) | 0.307 | 6.46  (-10.19 to 23.11) | 0.438 |
| Log DTF (%), mean (SD) | 3.65  (0.64) | 3.50  (0.58) | 0.15  (-0.20 to 0.51) | 0.389 | 0.11  (-0.24 to 0.47) | 0.531 |
| MIP cmH20, mean (SD) | 35.57  (11.90) | 29.71  (11.15) | 5.85  (-1.18 to 12.89) | 0.101 | 5.83  (-1.53 to 13.19) | 0.117 |
| PEF (L/min), mean (SD) | 83.20  (39.57) | 75.37  (34.08) | 7.83  (-16.18 to 31.85) | 0.513 | 7.80  (-16.59 to 32.19) | 0.521 |
| Extubation Failure, n (%) | 7.0  (29.2%) | 8.0  (32.0%) | 0.88  (0.26 to 2.95) | 0.83 | 0.99  (0.29 to 3.40) | 0.981 |
| SBT failure, median (IQR) | 0.0  (0.0 to 1.0) | 1.0  (0.0 to 1.0) | 0.47  (0.16 to 1.37) | 0.165 | 0.55  (0.18 to 1.64) | 0.283 |
| Time to extubation, median (IQR) | 8.0  (5.0 to 10.5) | 8.5  (5.0 to 13.5) | 1.28  (0.75 to 2.21) | 0.367 | 1.36  (0.77 to 2.38) | 0.288 |
| Days free of MV at d 28, median (IQR) | 19.5  (4.0 to 22.5) | 21.0  (13.0 to 23.0) | 0.67  (0.27 to 1.68) | 0.396 | 0.68  (0.26 to 1.76) | 0.430 |
| Tracheostomy, n (%) | 5.0  (18.5%) | 4.0  (15.4%) | 1.25  (0.30 to 5.28) | 0.762 | 1.33  (0.31 to 5.72) | 0.702 |
| ICU lOS, mean (SD) | 14.89  (9.52) | 18.21  (12.83) | -3.32  (-9.38 to 2.73) | 0.276 | -4.60  (-10.93 to 1.73) | 0.151 |

CI Confidence interval ; DTF Diaphragm thickening fraction ; SD Standard deviation ; MIP Maximal Inspiratory Pressure ; PEF Peak Expiratory Flow ; SBT Spontaneou Breathing Trial ; MV Mechanical Ventilation ; ICU Intensive Care Unit ; LOS Length of Stay
