# Supplementary material for: Transcutaneous electrical diaphragmatic stimulation in mechanically ventilated patients: a randomised study
Source: Crit Care. 2023 Aug 30;27:338. doi: 10.1186/s13054-023-04597-1 (PMC10469422; doi:10.1186/s13054-023-04597-1)
Supplement: Supplementary file 3 — Additional file 3. Intention to treat analysis with and without adjustment [file 13054_2023_4597_MOESM3_ESM.docx]

ESM 2 Intention to treat analysis with and without adjustement

|  |  |  | Non adjusted Intent to Treat Analysis | | Adjusted analysis Intent to Treat Analysis | |
| --- | --- | --- | --- | --- | --- | --- |
|  | TEDS Group | Sham Group | Estimate (95%CI) | p-value | Estimate (95%CI) | p-value |
| Primary outcome |  |  |  |  |  |  |
| DTF>30%, n (%) | 16.0  (66.7%) | 13.0  (54.2%) | 1.48  (0.46 to 4.76) | 0.512 | 1.55  (0.47 to 5.10) | 0.472 |
| DTF>20%, n (%) | 22.0  (91.7%) | 20.0  (83.3%) | 1.63  (0.29 to 9.21) | 0.578 | 1.61  (0.28 to 9.25) | 0.591 |
| Secondary outcomes |  |  |  |  |  |  |
| DTF (%), mean (SD) | 47.46  (34.19) | 38.67  (23.90) | 6.10  (-11.93 to 24.13) | 0.497 | 6.86  (-11.03 to 24.75) | 0.442 |
| Log DTF (%), mean (SD) | 3.65  (0.64) | 3.50  (0.58) | 0.11  (-0.33 to 0.55) | 0.621 | 0.12  (-0.32 to 0.56) | 0.585 |
| MIP cmH20, mean (SD) | 35.57  (11.90) | 29.71  (11.15) | 2.49  (-5.43 to 10.41) | 0.529 | 2.77  (-4.87 to 10.41) | 0.469 |
| PEF (L/min), mean (SD) | 83.20  (39.57) | 75.37  (34.08) | 3.61  (-23.02 to 30.23) | 0.784 | 3.58  (-21.78 to 28.94) | 0.776 |
| Extubation Failure, n (%) | 7.0  (29.2%) | 8.0  (32.0%) | 0.88  (0.26 to 2.95) | 0.83 | 0.88  (0.26 to 3.00) | 0.836 |
| SBT failure, median (IQR) | 0.0  (0.0 to 1.0) | 1.0  (0.0 to 1.0) | 0.53  (0.19 to 1.50) | 0.233 | 0.55  (0.19 to 1.56) | 0.259 |
| Time to extubation, median (IQR) | 8.0  (5.0 to 10.5) | 8.5  (5.0 to 13.5) | 1.34  (0.78 to 2.30) | 0.285 | 1.36  (0.79 to 2.34) | 0.262 |
| Days free of MV at d 28, median (IQR) | 19.5  (4.0 to 22.5) | 21.0  (13.0 to 23.0) | 0.71  (0.29 to 1.75) | 0.455 | 0.78  (0.31 to 1.92) | 0.583 |
| Tracheostomy, n (%) | 5.0  (18.5%) | 4.0  (15.4%) | 1.31  (0.31 to 5.51) | 0.715 | 1.29  (0.30 to 5.48) | 0.729 |
| ICU lOS, mean (SD) | 14.89  (9.52) | 18.21  (12.83) | -3.07  (-9.03 to 2.89) | 0.306 | -3.40  (-9.43 to 2.63) | 0.264 |

CI Confidence interval ; DTF Diaphragm thickening fraction ; SD Standard deviation ; MIP Maximal Inspiratory Pressure ; PEF Peak Expiratory Flow ; SBT Spontaneou Breathing Trial ; MV Mechanical Ventilation ; ICU Intensive Care Unit ; LOS Length of Stay
